# Soil Bacterial Community in the Multiple Cropping System Increased Grain Yield Within 40 Cultivation Years

**DOI:** 10.3389/fpls.2021.804527

**Published:** 2021-12-20

**Authors:** Tao Chen, Ruiwen Hu, Zhongyi Zheng, Jiayi Yang, Huan Fan, Xiaoqiang Deng, Wang Yao, Qiming Wang, Shuguang Peng, Juan Li

**Affiliations:** ^1^College of Agronomy, Hunan Agricultural University, Changsha, China; ^2^Hunan Tobacco Company Chenzhou Branch, Chenzhou, China; ^3^Hunan Tobacco Company Zhangjiajie Branch, Zhangjiajie, China; ^4^College of Bioscience and Biotechnology, Hunan Agricultural University, Changsha, China; ^5^Hunan Tobacco Monopoly Bureau, Changsha, China

**Keywords:** multiple cropping system, grain yield, soil properties, soil bacterial community, Acidobacteria, molecular ecology networks

## Abstract

The shortage of land resources restricts the sustainable development of agricultural production. Multiple cropping has been widely used in Southern China, but whether the continuous planting will cause a decline in soil quality and crop yield is unclear. To test whether multiple cropping could increase grain yield, we investigated the farmlands with different cultivation years (10–20 years, 20–40 years, and >40 years). Results showed that tobacco-rice multiple cropping rotation significantly increased soil pH, nitrogen nutrient content, and grain yield, and it increased the richness of the bacterial community. The farmland with 20–40 years of cultivation has the highest soil organic carbon (SOC), ammonium nitrogen, and grain yield, but there is no significant difference in the diversity and structure of the bacterial community in farmlands with different cultivation years. The molecular ecological network indicated that the stability of the bacterial community decreased across the cultivation years, which may result in a decline of farmland yields in multiple cropping system> 40 years. The Acidobacteria members as the keystone taxa (Zi ≥ 2.5 or Pi ≥ 0.62) appeared in the tobacco-rice multiple cropping rotation farmlands, and the highest abundance of Acidobacteria was found in the farmland with the highest SOC and ammonium nitrogen content, suggesting Acidobacteria *Gp4, GP7, GP12*, and *GP17* are important taxa involved in the soil carbon and nitrogen cycle. Therefore, in this study, the multiple cropping systems for 20 years will not reduce the crop production potential, but they cannot last for more than 40 years. This study provides insights for ensuring soil quality and enhancing sustainable agricultural production capacity.

## Highlights

- Multiple cropping can improve soil carbon and nitrogen, and increase crop yield for 20–40 years.
- Decreased bacterial community stability in multiple cropping system for long cultivation years (>40 years) could result in decrease in yield of farmland.
- *Acidobacteria* subgroups (e.g., *Gp4* and *GP17*) respond sensitively to the changes of soil carbon and nitrogen.

## Introduction

The multiple cropping system increases crop diversity, makes full use of limited soil resources, to a certain extent alleviates the shortage of cultivated land resources in agricultural production, and also guarantees food security (Yang et al., 2015; Xu et al., 2019b). Tobacco-rice multiple cropping rotation, that is, planting tobacco in spring and rice in autumn on the same farmland, is one of the main double-cropping systems in southern China (Hu et al., 2021). It not only ensures crop security production but also improves the income of farmers and effectively alleviates the social problems of rural laborers moving to cities and no production activities in cultivated land due to insufficient income (Xu et al., 2019a). Different cropping systems not only lead to differences in soil physical, chemical, and biological properties (Viaud et al., 2018; Wu et al., 2021) but also affect crop yield and quality (Caviglia et al., 2011), as well as have important implications for greenhouse gas emissions (Zhang, 2019). Under multiple cropping system, different fertilization methods, crop residue management, and rhizosphere microecosystem mediated by root exudates lead to differences in soil nutrient cycle and microbial community (Arcand et al., 2016; Li et al., 2017; Guyonnet et al., 2018; Zhao et al., 2021).

The paddy field needs flooding, and rice planting on the same farmland for many years will cause the occurrence of soil gleization (Liu et al., 2015). Studies have pointed out that the gleization of rice fields has low effective nutrient content, poor biological activity, and serious diseases, which are not conducive to the growth of rice, and the yield is severely reduced (Yuan et al., 2020). Continuous agricultural planting will reduce the diversity of soil bacterial community, change the community composition, find the proliferation of harmful bacteria in the soil with serious diseases in continuous cropping farm systems, and reduce the beneficial bacteria (Wu et al., 2017; Hu et al., 2019). Increasing cultivation years changed the structure and diversity of soil bacterial and fungal communities in farmland, and the long-term fertilization increased soil organic carbon (SOC) storage and macroaggregate content (Zhang et al., 2019; Li et al., 2020), but these studies focused on farmland whose cultivation years generally spanned about 20 years, and there were fewer studies on the changing trends of soil bacterial community in multiple cropping farmlands with more than 40 years of cultivation.

As an important part of the soil, microorganisms are very sensitive to soil environmental changes, such as pH, SOC, nutrient, water, and temperature (Griffiths and Philippot, 2013; Hartmann et al., 2015; Barcenas-Moreno et al., 2016; Frac et al., 2018). Soil microorganisms play an important role in the decomposition of soil organic matter, material transformation, energy transfer, and other ecological processes (Frey et al., 2013; Viggi et al., 2014). The interaction between rhizosphere microorganisms and plants can promote plant growth, enhance plant stress resistance, and help plants resist diseases and abiotic stress, which is called “the second genome of plants” (Mendes et al., 2013; Rodriguez et al., 2019). The energy and nutrients required by microorganisms mainly come from soil carbon and nitrogen, so the change of soil carbon and nitrogen concentration will affect the structure and composition of the microbial community (Yao et al., 2021b), leading to the transformation of its function in the soil ecosystem. Ammonium and nitrate nitrogen are the two main inorganic nitrogen that plants can directly absorb and utilize, both of which affect the diversity and structure of the microbial community (Li et al., 2019b). However, different forms of soil nitrogen have different effects on bacterial and fungal communities: nitrate drives bacterial communities, and ammonium nitrogen affects fungal communities (Li et al., 2021b).

A large number of different bacterial taxa were present in the soil, yet <1% of bacteria from the natural environment could be cultured by the conventional culture techniques (Chaudhary et al., 2019). In recent years, researchers have paid increasing attention to the role played by Acidobacteria taxa in participating in soil nutrient cycling and interactions with plants (Kielak et al., 2016). However, due to the difficulty of isolation and culture of Acidobacteria, the understanding of its physiological characteristics and function is limited (Eichorst et al., 2011). Acidobacteria exists in various habitats, and the quantity of Acidobacteria in soil accounts for about 20–50% of the total bacterial community, which plays an important role in the construction of soil ecosystem (Jones et al., 2009). Studies have reported that Acidobacteria is sensitive to soil pH and nitrogen deposition levels, and different Acidobacteria subgroups have different responses to pH and nitrogen levels (Liu et al., 2017; Yao et al., 2021a). At the same time, different soil habitats will also lead to differences in the distribution of Acidobacteria (Naether et al., 2012).

The purpose of this study was to clarify the differences in soil properties and crop yields in multiple cropping farmlands under different cultivation years and to evaluate the relationship between the changes of bacterial community diversity and composition and soil properties. We hypothesized that the differences in soil physicochemical properties and bacterial community in multiple cropping farmlands with different cultivation years caused the changes in crop yields. Our research attempts to explain the change of crop yield from the perspective of bacterial interaction and community stability, which will help to understand the importance of the interaction between soil-crop-microbial in the farmland ecosystem.

## Materials and Methods

### Soil Sample Collection and Farmland Management

On November 5, 2020, after the harvest of rice in the tobacco-rice multiple cropping rotation farmlands, soil samples were collected from farmlands with different cultivation years (Table 1). All samples were collected from Guiyang County, Chenzhou City, Hunan Province, China. The average annual temperature in Guiyang County from 2018 to 2020 is 17.6°C, the annual average precipitation is 1,568 mm, the annual average frost-free period is about 281 days, and the annual sunshine hours are 1,475 h. According to the principle of random, multisite mixed soil sample collection, a shovel was used to collect 6 soil samples at each sampling site, and 3 of them were completely mixed into 1 soil sample, so 2 soil samples per sampling site, a total of 16 sampling sites, and 32 soil samples. The impurities, such as roots, stones, and residual fertilizer, were removed from the soil. Then, we divided each soil sample into two parts and put them into sterile ziplock bags. One of the bags of soil was frozen in liquid nitrogen, then put in an icebox and transported back to the laboratory, and stored in an ultralow temperature refrigerator at −80°C for DNA extraction. Another bag of soil was stored at room temperature to determine the physical and chemical properties of the soil.

**Table 1:** Sampling sites, altitude, and cultivation years of tobacco-rice multiple cropping rotation farmlands.

**Planting period (years)**	**Treatment**	**Sampling sites (Guiyang County, Chenzhou City, Hunan Province, China)**	**Altitude (m)**	**Cultivation years**
0	CK	Heping Town (25°59′37″N, 112°38′40″E)	284	0
		Zhangshi Town (25°50′25″N, 112°46′27″E)	200	0
		Renyi Town (25°46′41″N, 112°40′07″E)	232	0
		Renyi Town (25°46′41″N, 112°40′07″E)	232	0
10–20	Y10	Aoquan Town (25°55′54″N, 112°35′16″E)	263	17
		Zhangshi Town (25°50′25″N, 112°46′27″E)	200	16
		Renyi Town (25°46′41″N, 112°40′07″E)	232	13
		Heping Town (25°59′37″N, 112°38′40″E)	284	15
20–40	Y20	Aoquan Town (25°55′54″N, 112°35′16″E)	263	26
		Renyi Town (25°46′41″N, 112°40′07″E)	232	31
		Heping Town (25°59′37″N, 112°38′40″E)	284	29
		Heping Town (25°59′37″N, 112°38′40″E)	284	33
>40	Y40	Renyi Town (25°46′41″N, 112°40′07″E)	232	57
		Zhangshi Town (25°50′25″N, 112°46′27″E)	200	61
		Wutong Town (25°46′24″N, 112°41′52″E)	230	51
		Wutong Town (25°46′24″N, 112°41′52″E)	230	47

In rice monoculture farmland, rice is planted in April each year and then fallow. The tobacco-rice multiple cropping rotation farmlands is planted with flue-cured tobacco in March and rice in August each year. All sampling sites (farmlands) were fertilized according to the same local fertilization scheme: the total fertilization amount used for flue-cured tobacco planting was 165 kg N ha^−1^, 165 kg P_2_O_5_ ha^−1^, and 420 kg K_2_O ha^−1^, and the total fertilization amount used for rice planting was 150 kg N ha^−1^, 75 kg P_2_O_5_ ha^−1^, and 120 kg K_2_O ha^−1^. During the planting period of flue-cured tobacco, nitrogen fertilizer is applied according to base fertilizer: topdressing = 5:5, phosphorus fertilizer is applied as base fertilizer at one time, and potassium fertilizer is applied according to base fertilizer: topdressing = 3:7. In the rice planting period, nitrogen fertilizer is applied according to base fertilizer: tiller fertilizer: panicle fertilizer = 5:3:2, phosphorus fertilizer is applied as base fertilizer at one time, and potassium fertilizer is applied according to base fertilizer: tiller fertilizer = 5:5. The tobacco planted variety was Yunyan 87, and the rice planted variety was Qianfengyou 877 (hybrid rice). All other farmland management measures were consistent.

### Soil Physical and Chemical Properties

The measurement methods of soil physical and chemical properties followed the protocols detailed in the study by Bao (2000). Soil pH was measured in 1:2.5 mixtures of soil and deionized water with a pH meter (PHS-3C, Lei-ci, Shanghai, China). SOC was determined by potassium dichromate (K_2_Cr_2_O_7_) and sulfuric acid (H_2_SO_4_) oxidation and titration. Ammonium-N (${\text{NH}}_{4}^{+}$-N) and nitrate-N (${\text{NO}}_{3}^{-}$-N) were extracted with the ratio of 5 g fresh soil to 50 ml 2 M potassium chloride. The contents of ${\text{NO}}_{3}^{-}$-N and ${\text{NH}}_{4}^{+}$-N were analyzed by a continuous flow analytical system (San++ system, Skalar, Holland).

### Grain Yield Measurement

The grain yield measurement of the tobacco-rice multiple cropping rotation farmlands was completed on October 29, and the grain yield measurement of the rice monoculture farmland was on July 20. Grain yield was determined using a 5 m^2^ area in the center of each sampling site and adjusted to 14% of the standard water content (Huang et al., 2020).

### DNA Extraction, PCR Amplification, and Sequencing

Genomic DNA was extracted from fresh samples of 1.0 g with the Fast DNA® SPIN Kit for Soil (MP Biomedicals, Santa Ana, CA, USA). The V4–V5 region of bacterial 16S rRNA was amplified together with a specific Illumina adapter and barcode sequence primer pair 515F (5′-GTGCCAGCMGCCGCGGTAA-3′) and 806R (5′-GGACTACHVGGGTWTCTAAT-3′) (Gu et al., 2019). PCR was conducted in 25 μl mixtures containing 1.0 μl (~25 ng DNA) template, 12.5 μl PCR Premix, 2.5 μl of forward/reverse primers, and DNase-RNase-free deionized water to adjust the volume. The PCR procedure consisted of 98°C denaturing for 30 s, followed by 32 cycles of 98°C for 10 s, 54°C for 30 s, 72°C for 45 s, and a final step of 72°C extensions for 10 min. After purification and quantification, the PCR product constructed that the library sequencing was performed on the Illumina NovaSeq PE250 platform (LC-Bio Technology Co., Ltd., Hang Zhou, Zhejiang Province, China).

### Sequencing Data Analysis

The sequence was processed, the primers on the Galaxy pipeline were removed (http://mem.rcees.ac.cn:8080/), Btrim was used for low-quality sequence filtering, the low-quality reads with a quality control score of <20 and a length of <200 bp were removed, and then Flash was used to splice the forward and reverse sequences together and overlap by 20–250 bp to obtain high-quality sequences (Gu et al., 2019). Through the UPARSE classification operation, operational taxonomic unit (OTU) clustering is performed at a similar level of 97%, and the ribosomal database project (RDP) classifier is used to classify and annotate each sequence (Bacci et al., 2015). We obtained 45,637–73,902 clean sequences per sample and then randomly rarefied to 45,637 sequences per sample.

The bacterial community analysis includes alpha diversity analysis (Shannon, Simpson, Pielou evenness, and Chao1) and beta diversity analysis, including non-metric multidimensional scaling (NMDS) analysis, principal component analysis (PCA), and community dissimilarity test based on the Bray–Curtis distance matrix (ANOSIM, MRPP, and ADONIS), and all the above analyses were calculated using “vegan” and “stats” packages and plotted using the “ggplot2” package (Oksanen et al., 2006; Gómez-Rubio, 2017). A Venn diagram was obtained using the “VennDiagram” package. Pearson's correlation between bacteria and soil properties was calculated using the Statistical Package for Social Science (SPSS, IBM, New York, USA) 19.0 for Windows. To determine the specific differential taxa of bacterial community composition in different treatments, the linear discriminant analysis (LDA) Effect Size (LEfSe) was completed on the Galaxy pipeline (http://mem.rcees.ac.cn:8080/), and the threshold on the logarithmic LDA score was 3.0. Pairwise Pearson's correlation matrix of the edaphic factors and Acidobacteria subgroups was completed with the “corrplot” package (Yang et al., 2021). At the *p* < 0.05 level, the least significant difference (LSD) method was used for ANOVA.

### Network Construction and Analysis

Based on the random matrix theory (RMT), a phylogenetic molecular ecological network (pMEN) was constructed by using the 16S rRNA sequencing data (Deng et al., 2012). The network was constructed using at least 8 detected OTUs out of the 10 replicates in this study. Network construction and analysis were executed on the MENA pipeline of the Oklahoma University IEG (http://ieg4.rccc.ou.edu/mena/). The first step of network construction is to upload the standardized OTU sequence data. Second, the relative abundance and the OTU of different samples based on Pearson's correlation coefficient are analyzed and converted into a similarity matrix, which can measure the degree of agreement. Third, using the RMT-based network method, the adjacency matrix is obtained by applying an appropriate threshold to the similarity matrix, thereby defining the distance between each pair of nodes (Zhou et al., 2010). Finally, a network containing nodes and links is obtained, and some network topology characteristics are generated. The constructed network is visualized by Gephi 0.9.2-beta. To determine the topological role of each node in the network, a Zi-Pi chart display based on the within-module connectivity (Zi) and among-module connectivity (Pi) values was used. Module hubs, connectors, and network hubs have been proposed as potential keystone taxa due to their important roles in network topology.

## Results

### Soil Properties and Grain Yield

Compared with rice monoculture (CK treatment), the tobacco-rice multiple cropping rotation (Y10, Y20, and Y40 treatments) significantly improved soil pH value, SOC content, ammonium nitrogen content, and grain yield (*p* < 0.05, Table 2). Comparing tobacco-rice multiple cropping rotation farmlands with different cultivation years, the soil pH value, SOC content, ammonium nitrogen content, and grain yield were the highest in the Y20 treatment, and the nitrate nitrogen content was the highest in the Y40 treatment. The content of SOC and ammonium nitrogen affected the grain yield, while nitrate nitrogen had no effect.

**Table 2:** Comparison of soil physical and chemical properties and grain yield in different cultivation years of tobacco-rice multiple cropping rotation farmlands.

**Treatment**	**pH**	**SOC (g/kg)**	**${\text{NH}}_{4}^{+}$-N (mg/kg)**	**${\text{NO}}_{3}^{-}$-N (mg/kg)**	**Grain yield (kg/ha)**
CK	7.22 b	25.83 c	21.87 d	2.05 b	7084.13 c
Y10	7.51 a	26.39 c	27.29 b	1.15 d	7197.49 b
Y20	7.54 a	33.01 a	28.94 a	1.57 c	7389.58 a
Y40	7.53 a	30.61 b	25.32 c	2.66 a	7221.09 b

*Results are means of replicates (n = 8). Different lowercase letters in the same column of each treatment are significantly different at the 0.05 probability level. SOC, soil organic carbon; ${\text{NH}}_{4}^{+}$-N, ammonia-N; ${\text{NO}}_{3}^{-}$-N, nitrate-N*.

### Diversity and Structure of Soil Bacterial Community

**Figure 1:**
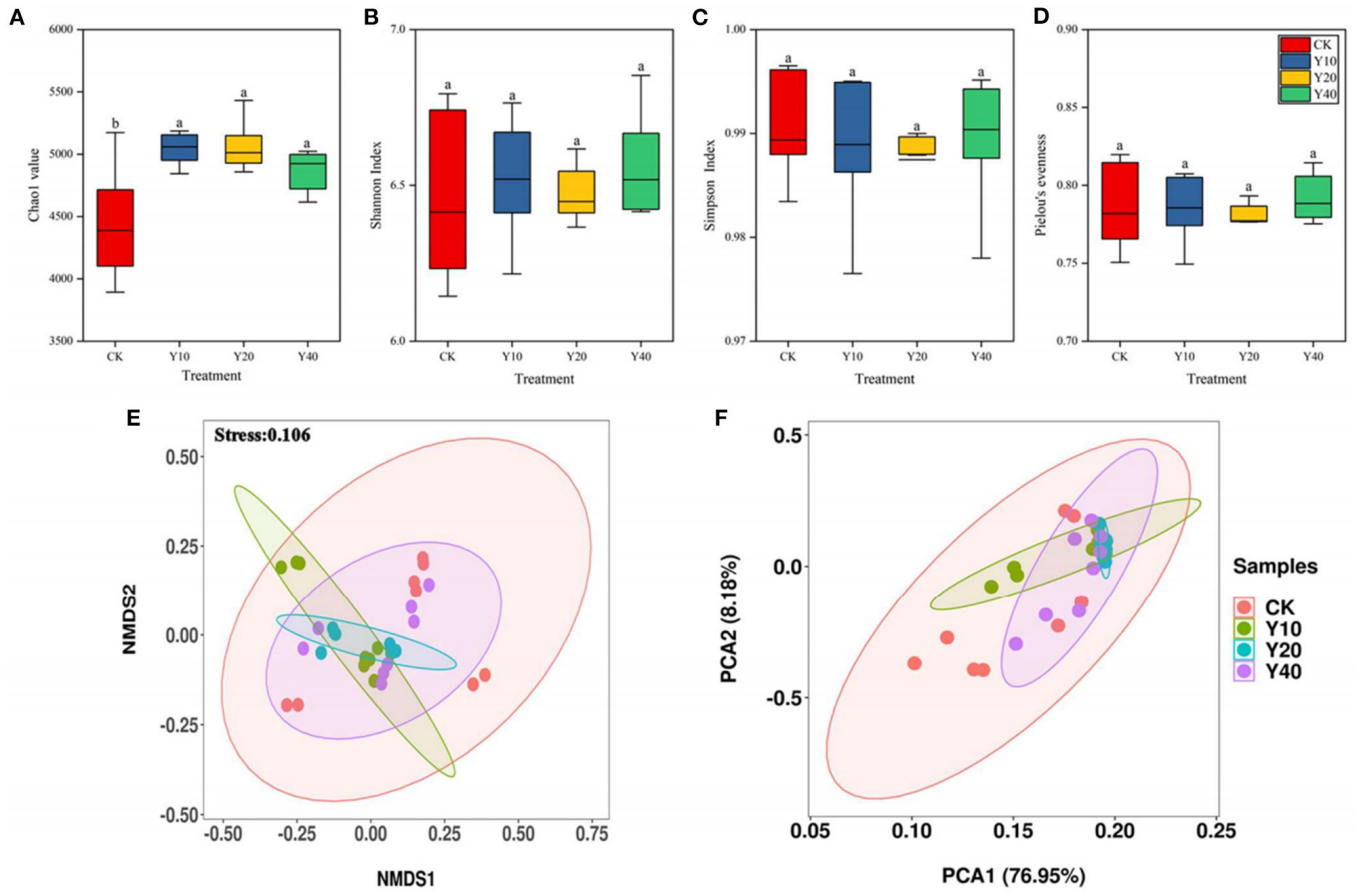
Alpha diversity of 16S rRNA gene sequencing data among groups CK, Y10, Y20 and Y40. **(A)** Chao1 value, **(B)** Shannon Index, **(C)** Simpson Index, **(D)** Pielou's evenness. Results are means of eight replicates. Different letter means the significant difference (*P* < 0.05). The non-metric multidimensional scaling (NMDS) **(E)** and principal component analysis (PCA) **(F)** plots of soil bacterial community beta diversity.

The alpha diversity of soil bacterial communities in different treatments generally showed that there is no significant difference among treatments (Figures 1A–D), only Chao1 value was significantly lower in CK treatment than in Y10, Y20, and Y40 treatments (*p* < 0.05). Compared with rice monoculture farmland, a higher abundance of the soil bacterial community was observed in tobacco-rice multiple cropping rotation farmlands, but there is no significant difference in the alpha diversity of soil bacterial community in tobacco-rice multiple cropping rotation farmlands with different cultivation years.

The NMDS (Figure 1E) and PCA (Figure 1F) further confirmed that the bacterial community structure of the CK treatment and Y10, Y20, and Y40 treatments was separated clearly. The two main axes of PCA explained 72.08% of the variation, indicating that it can better represent the characteristics of microbial community composition, of which 61.61% of the variation is explained by PC1, and 10.47% of the variation is explained by PC2. The dissimilarity analysis (i.e., ADNOIS, ANOSIM, and MRPP) based on the Bray–Curtis distance further confirmed that there are significant differences in the soil bacterial community structure between rice monoculture farmland and tobacco-rice multiple cropping rotation farmlands (*p* < 0.05, Supplementary Table S1), while there were no significant differences in soil bacterial community structure between tobacco-rice multiple cropping rotation farmlands with different cultivation years.

### Composition of Soil Bacterial Community and Its Drivers

**Figure 2:**
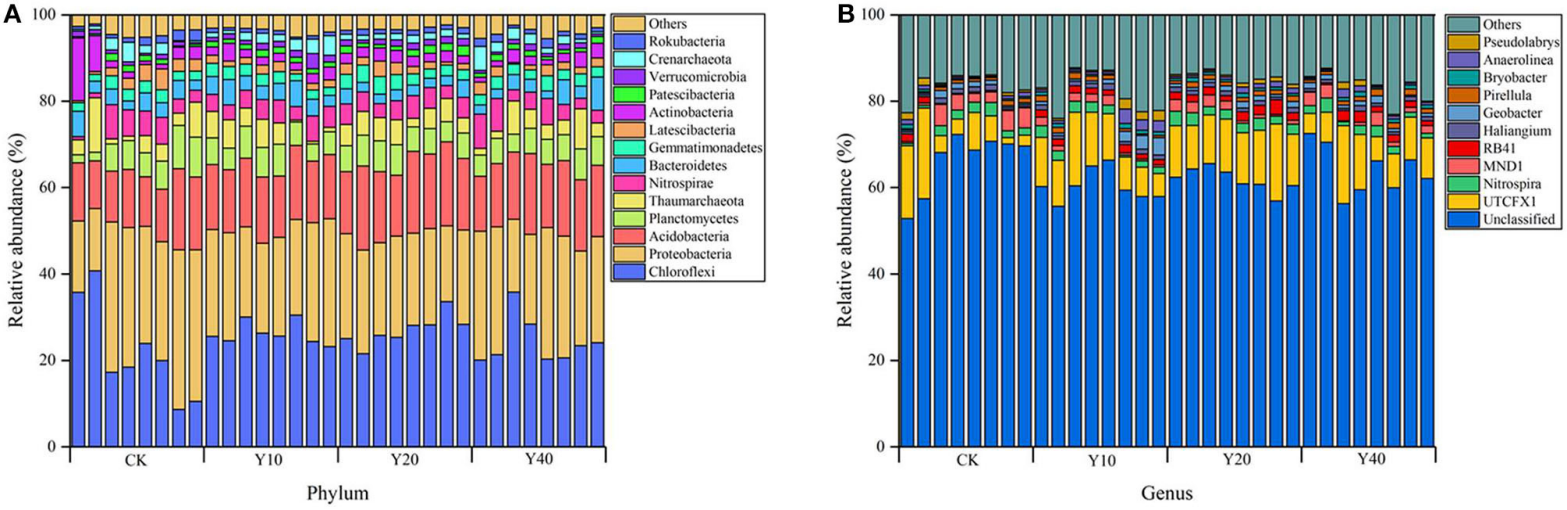
Soil bacterial community composition: **(A)** Relative abundance at phylum level; **(B)** Relative abundance at genus level. Among the bacterial communities in all samples, phylum and genera with an abundance of <1% were classified as “others.”

In terms of the taxonomic composition of soil bacterial communities at the phylum level, there were 57, 57, 54, and 59 different phyla in CK, Y10, Y20, and Y40 treatments, respectively. The bacterial communities in all treatments were mainly composed of Chloroflexi, Proteobacteria, and Acidobacteria, and their relative abundance accounted for more than half of the entire community (Figure 2A). It should be noted that the relative abundance of Proteobacteria in CK treatment was higher than that in multiple cropping treatments, while the relative abundance of Chloroflexi and Acidobacteria was lower than that in multiple cropping treatments. Tobacco-rice multiple cropping rotation showed a stronger symbiotic relationship between Chloroflexi and Acidobacteria. At the genus level, there are 720, 729, 694, and 751 different genera in CK, Y10, Y20, and Y40 treatments, respectively. *UTCFX1* is the genus with the highest relative abundance among all identified genera (Figure 2B). The mean relative abundance of *UTCFX1* in CK, Y10, Y20, and Y40 treatments were 7.99, 10.27, 12.43, and 9.39%, respectively. From the relative abundance of the heat map and the scale of the color change, the differences in the composition of the soil bacterial community at the genus level among the treatments can be observed more clearly (Supplementary Figure S1). The relative abundance of *MND1* and *Anaeromyxobacter* was higher in CK; *Pseudolabrys, Anaerolinea, Sideroxydans, Geobacter*, and *Haliangium* were higher in Y10; *Nitrospira, Gemmatimonas, RB41*, and *UTCFX1* were higher in Y20; and *Thiobacillus* was higher in Y40. In addition, according to the cluster analysis of the heat map, the treatments were divided into two groups. All tobacco-rice multiple cropping rotation farmlands (Y10, Y20, and Y40) were clustered together, and rice monoculture farmlands (CK) were grouped into one group separately, which is consistent with the results of NMDS.

**Figure 3:**
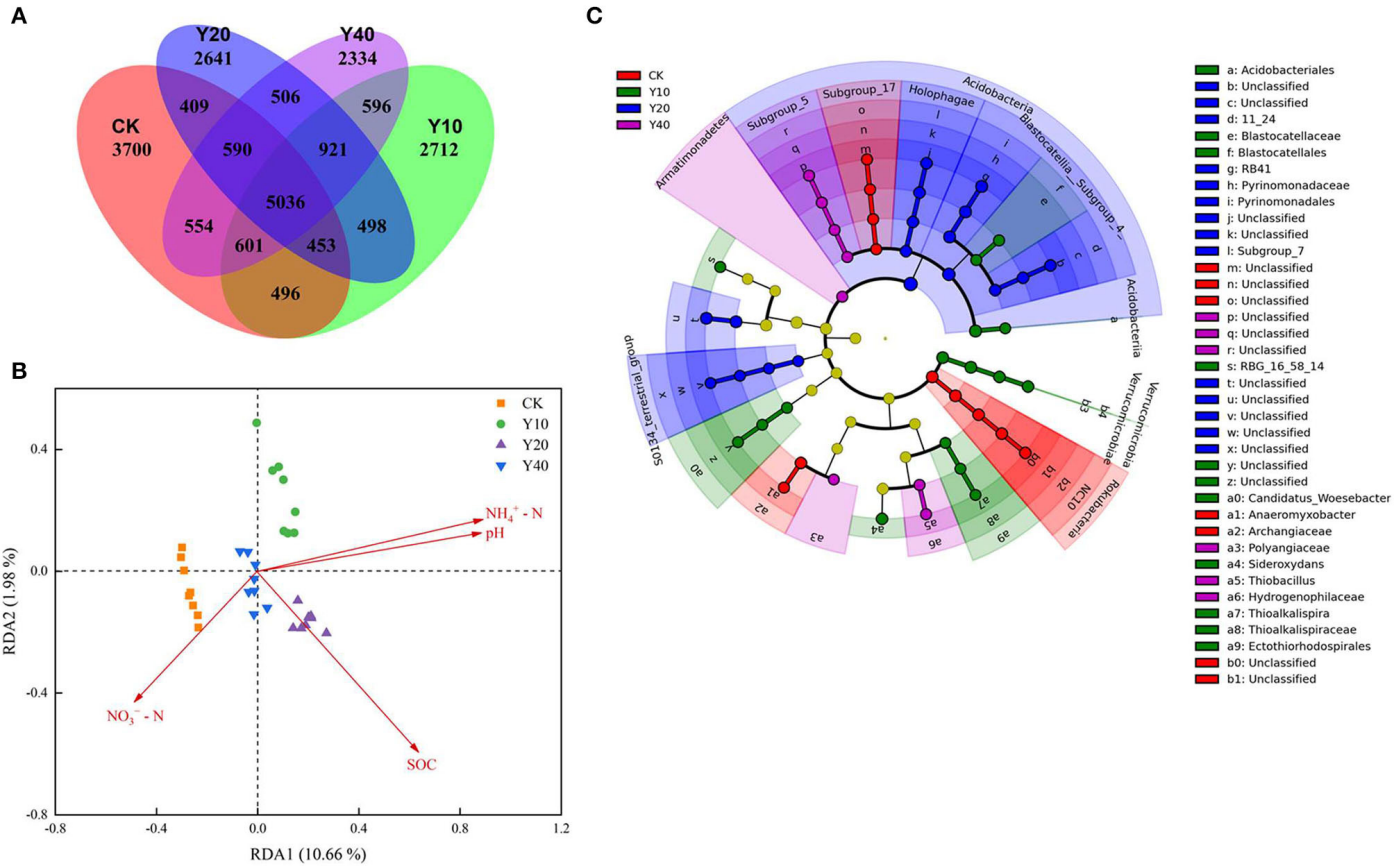
Venn diagrams showing the shared and unique OTUs **(A)** and redundancy analysis shows soil properties drive the bacterial community **(B)**. Linear discriminant analysis Effect Size (LEfSe) identified the size of differentiation in bacterial abundances between different treatments with a threshold value of 3.0 **(C)**. SOC, Soil organic carbon; ${\text{NH}}_{4}^{+}$-N, Ammonia-N; ${\text{NO}}_{3}^{-}$-N, Nitrate-N.

The Venn diagram showed that the CK treatment had more unique OTUs (Figure 3A), and with the increase in the cultivation years of tobacco-rice multiple cropping rotation farmlands, the number of unique OTUs in the Y20 and Y40 treatments gradually decreased. The environmental factors driving the change of bacterial community were revealed by the redundancy analysis (RDA) analysis and the envfit analysis. The results of the RDA plot (Figure 3B) and the envfit analysis (Supplementary Table S2) show that soil pH, ammonium nitrogen, and nitrate nitrogen were the environmental variables that significantly affected the changes of soil bacterial communities (*p* < 0.05), and the four environmental variables cumulatively explain 14.98% of the variation of the bacterial community (Supplementary Table S3).

Through the LEfSe analysis (threshold value of 3.0), the specificity in bacterial community composition among different treatments was analyzed, and 53 bacterial taxa (Supplementary Table S4) showed significant differences (*p* < 0.05). The cladogram (Figure 3C) showed that at the phylum level, Rokubacteria was highly enriched in the CK treatment, Verrucomicrobia was highly enriched in the Y10 treatment, Acidobacteria was highly enriched in the Y20 treatment, and Armatimonadetes was highly enriched in the Y40 treatment. Pearson's correlation analysis (Table 3) of the bacterial phyla that were significantly enriched in each treatment and soil properties showed that Rokubacteria was significantly positively correlated with soil nitrate nitrogen; Verrucomicrobia was significantly positively correlated with soil ammonium nitrogen and significantly negatively correlated with nitrate nitrogen; and Acidobacteria and Armatimonadetes were significantly positively correlated with soil pH, SOC, and ammonium nitrogen. The soil characteristics of the highest pH, SOC, and ammonium nitrogen in tobacco-rice multiple cropping rotation farmlands with a cultivation period of 20–40 years drive more Acidobacteria to survive in the soil.

**Table 3:** Pearson's correlation between soil properties and phylum abundance in the bacterial community.

**Phylum**	**pH**		**SOC**		**${\text{NH}}_{4}^{+}$-N**		**${\text{NO}}_{3}^{-}$-N**	
	**Pearson**	***p***	**Pearson**	***p***	**Pearson**	***p***	**Pearson**	***p***
*Armatimonadetes*	0.644	**0**	0.434	**0.013**	0.602	**0**	−0.081	0.661
*Acidobacteria*	0.492	**0.004**	0.505	**0.003**	0.515	**0.003**	−0.059	0.750
*Verrucomicrobia*	0.293	0.104	0.090	0.624	0.447	**0.010**	−0.452	**0.009**
*Rokubacteria*	−0.245	0.177	0.089	0.629	−0.325	0.070	0.374	**0.035**

*The bold numbers indicate a significant correlation at the level of p < 0.05. “0” means the p < 0.001. SOC, soil organic carbon; ${\text{NH}}_{4}^{+}$-N, ammonia-N; ${\text{NO}}_{3}^{-}$-N, nitrate-N*.

### Relative Abundance of Acidobacteria Subgroups and Its Relationship With Edaphic Factors

**Figure 4:**
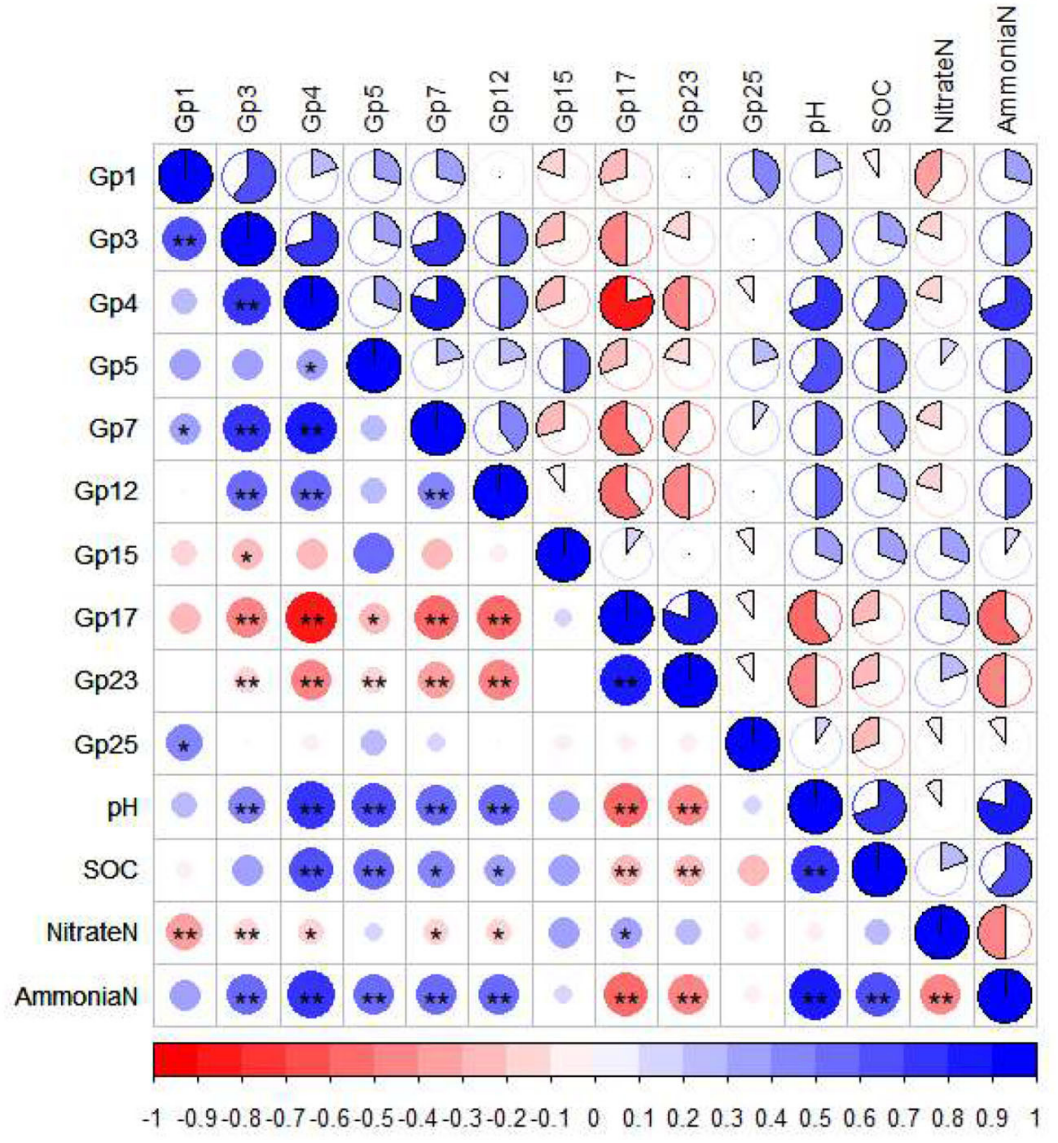
Pairwise Pearson's correlation matrix of the edaphic factors and *Acidobacteria* subgroups was shown with heat map, * Significant at the *P* = 0.05 level, ** Significant at the *P* = 0.01 level. SOC, Soil organic carbon.

Since the farmland (Y20) with the highest SOC and soil ammonium nitrogen content had the most enrichment of Acidobacteria (Supplementary Table S4), we speculated that Acidobacteria plays an important role in the soil carbon and nitrogen cycle. To further understand the distribution of Acidobacteria in farmland with different cultivation years, we compared the relative abundance of 21 different Acidobacteria subgroups (Table 4). In the CK treatment, the relative abundance of Acidobacteria *GP17* was significantly higher than other treatments (*p* < 0.05). In the Y10 treatment, the relative abundance of Acidobacteria *GP1* was significantly higher than other treatments (*p* < 0.05). In the Y20 treatment, the relative abundance of Acidobacteria *GP4* and Acidobacteria *GP7* was significantly the highest (*p* < 0.05). The relative abundance of Acidobacteria *GP15* was significantly highest in the Y40 treatment (*p* < 0.05). The heat map of the pairwise Pearson's correlation matrix (Figure 4) of edaphic factors and Acidobacteria subgroups shows that Acidobacteria *GP1* is significantly negatively correlated with soil nitrate nitrogen, Acidobacteria *GP3, GP4, GP5, GP7*, and *GP12* are significantly positively correlated with soil pH and ammonium nitrogen, and Acidobacteria *GP17* and *GP23* are significantly negatively correlated with soil pH, SOC, and ammonium nitrogen (*p* < 0.01). Noticeably, compared with rice monoculture, tobacco-rice multiple cropping rotation significantly increased soil pH (Table 2), and the Acidobacteria *GP17* abundance decreased significantly (*p* < 0.01), with a maximum decrease of 54.65%. The abundance of Acidobacteria *GP4* and *GP5* increased significantly (*p* < 0.01), with the highest increases of 152.05 and 101.08%, respectively.

**Table 4:** Percentage of Acidobacteria subgroup abundance relative to all bacterial communities.

***Acidobacteria*** **subgroups**	**CK**	**Y10**	**Y20**	**Y40**	**Statistics**
*Gp1*	0.007 b	0.068 a	0.020 b	0.007 b	*
*Gp2*	0.060 a	0.049 a	0.093 a	0.090 a	ns
*Gp3*	0.844 b	1.445 a	1.449 a	1.310 ab	*
*Gp4*	1.462 b	2.883 a	3.685 a	2.889 a	**
*Gp5*	0.278 b	0.464 a	0.521 a	0.559 a	**
*Gp6*	6.221 a	5.864 a	6.700 a	6.886 a	ns
*Gp7*	0.499 b	0.887 ab	1.210 a	0.820 ab	*
*Gp9*	0.079 a	0.028 a	0.051 a	0.098 a	ns
*Gp10*	0.318 a	0.276 a	0.310 a	0.283 a	ns
*Gp11*	0.180 a	0.140 a	0.177 a	0.165 a	ns
*Gp12*	0.005 b	0.021 a	0.019 a	0.017 a	*
*Gp13*	0.093 a	0.131 a	0.088 a	0.105 a	ns
*Gp15*	0.045 b	0.055 b	0.093 ab	0.135 a	*
*Gp17*	0.763 a	0.346 b	0.385 b	0.443 b	**
*Gp18*	0.500 a	0.489 a	0.259 a	0.276 a	ns
*Gp19*	0.003 a	0.006 a	0.006 a	0.004 a	ns
*Gp20*	0.032 a	0.035 a	0.035 a	0.041 a	ns
*Gp21*	0.058 a	0.061 a	0.058 a	0.041 a	ns
*Gp22*	1.582 a	0.932 a	1.310 a	0.992 a	ns
*Gp23*	0.006 a	0.002 ab	0.001 b	0.003 ab	*
*Gp25*	0.304 ab	0.401 a	0.246 b	0.326 ab	*
Others	0.283 a	0.580 a	0.360 a	0.360 a	ns
Total *Acidobacteria* community	13.621 b	15.163 ab	17.071 a	15.851 a	*

*Gp, subgroup. Results are the means of eight replicates. Different lowercase letters in the same line of each treatment are significantly different at the 0.05 probability level. ns, not significant at the p = 0.05 level*.

* *Significant at the p = 0.05 level*.

** *Significant at the p = 0.01 level*.

### RMT-Based Molecular Ecological Networks

**Figure 5:**
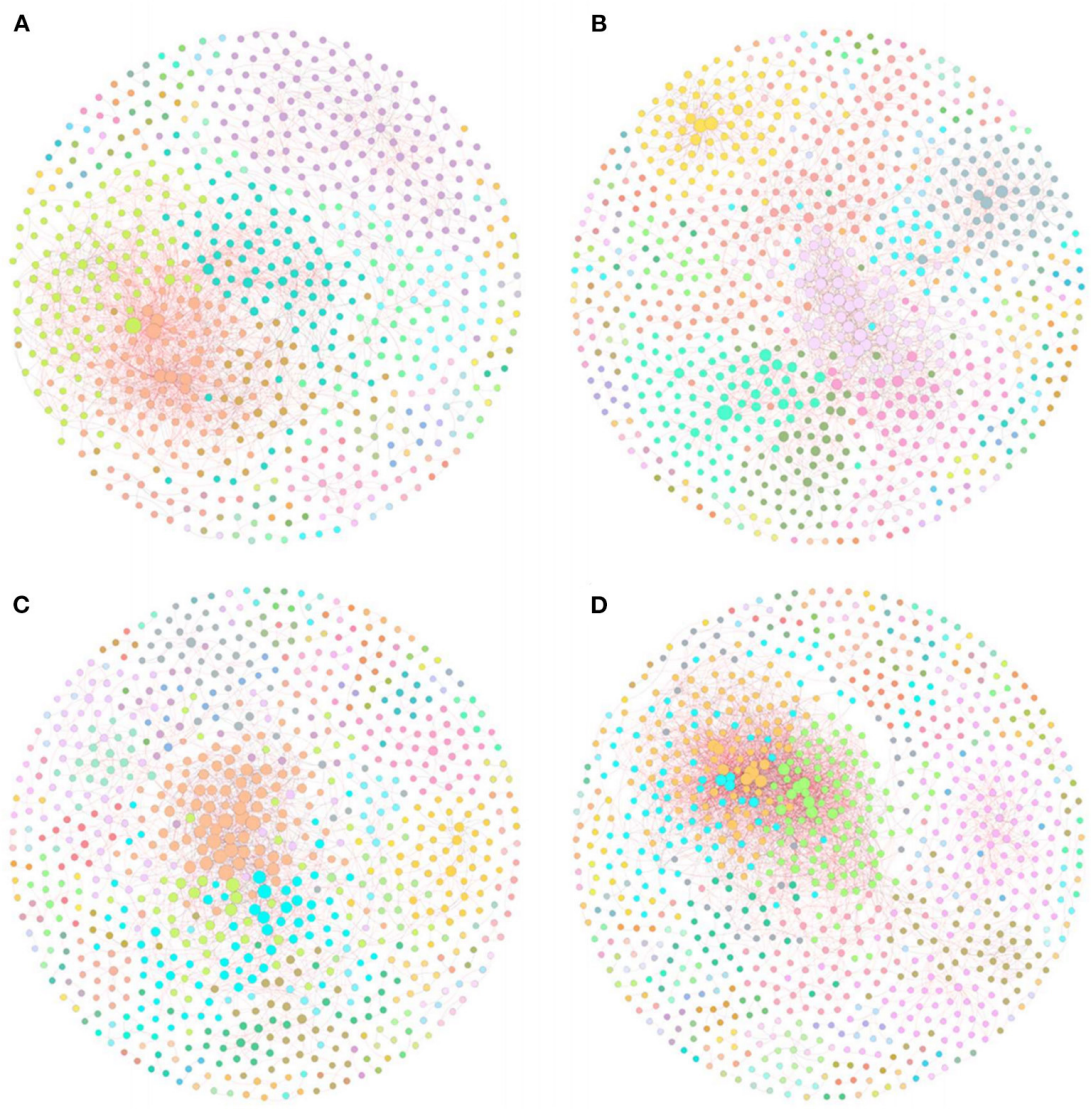
The RMT-based molecular ecological networks of CK **(A)**, Y10 **(B)**, Y20 **(C)**, Y40 **(D)**. In the networks, each node represents an OUT, and edges between nodes correspond to either positive (red) or negative (black) correlations. Modules were randomly colored.

To investigate the differences in bacterial interaction between rice monoculture farmland and tobacco-rice multiple cropping rotation farmlands with different cultivation years, four molecular ecological networks based on RMT were constructed using the 16s rRNA sequencing data of each treatment (Figure 5). The topological features of the molecular ecological networks (Table 5) showed that the connectivity distribution curves of all four networks fit well with the power-law model, with the *R*^2^ values of 0.818, 0.915, 0.913, and 0.839, respectively. Y10, Y20, and Y40 have more nodes than CK, but Y10 and Y20 have fewer links than CK, and Y40 has the most nodes and links (913 nodes and 3,680 links). Compared with other treatments, Y40 has the highest node, link, and average connectivity, as well as the lowest average path distance and harmonic geodetic distance, so its network is more complex, and the connection between bacteria was closer. However, Y40 has the lowest modularity, indicating that its network stability was poor, and it was more difficult to resist the interference of external environmental changes. The percentages of positive correlations in CK (80.03%), Y10 (70.96%), Y20 (67.25%), and Y40 (73.80%) were greater than the negative correlations, but the percentages of positive correlations in Y10, Y20, and Y40 treatments were lower than those of CK, indicating that tobacco-rice multiple cropping rotation would strengthen the competitive relationship between bacteria. We tried to clarify the relationship between edaphic factors and bacterial positive correlation by using the linear regression equation. The results showed that there is a significant negative correlation (*p* < 0.01) between soil ammonium nitrogen and the percentages of positive links (Supplementary Figure S2). The cooperative relationship between bacteria will weaken, and the competitive relationship will become stronger with the increase of soil ammonium nitrogen content.

**Table 5:** Major topological features of the molecular ecological networks of bacterial communities in CK, Y10, Y20, and Y40.

	**Network index**	**CK**	**Y10**	**Y20**	**Y40**
Empirical networks	Similarity threshold	0.970	0.960	0.960	0.960
	Total nodes	699	878	808	913
	Total links	2544	2455	2000	3680
	*R*^2^ of power law	0.818	0.915	0.913	0.839
	Positive links (%)	80.03	70.96	67.25	73.80
	Average connectivity	7.279	5.592	4.950	8.061
	Average path distance	5.949	6.238	5.838	5.167
	Harmonic geodesic distance	4.125	4.992	4.711	3.787
	Average clustering coefficient	0.197	0.245	0.249	0.182
	Modularity (no. of modules)	0.553 (64)	0.696 (90)	0.633 (100)	0.487 (114)

**Figure 6:**
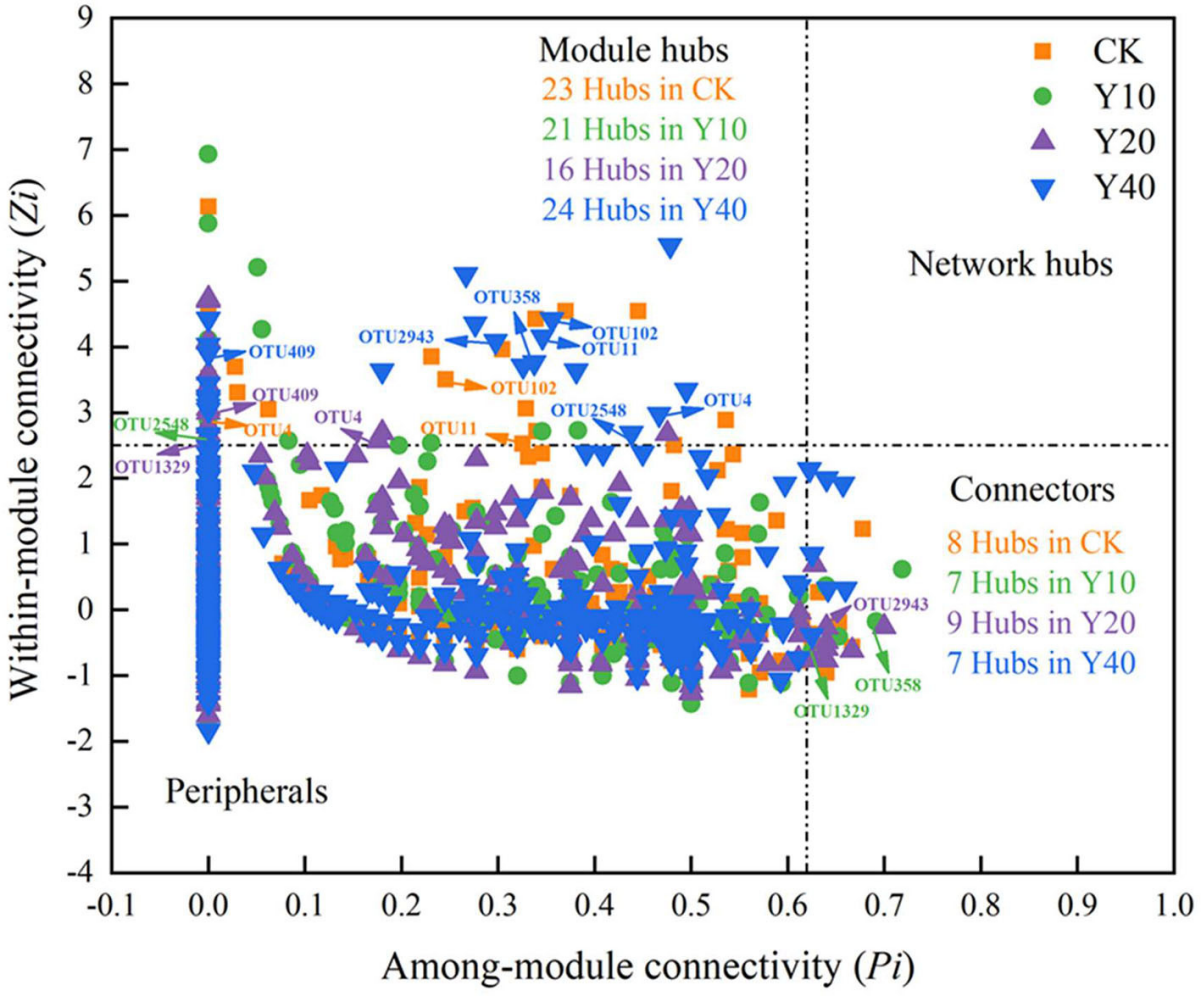
Each node was identification to classify the keystone taxa in molecular ecological networks of bacterial communities. Each symbol represents an OTU from the network of CK, Y10, Y20 and Y40. The value of Zi and Pi confirmed each OTU's topological role. Nodes were identified into four models depended on their Zi and Pi values: module hubs (Zi > 2.5 and Pi < 0.62), network hubs (Zi > 2.5 and Pi > 0.62), connectors (Zi < 2.5 and Pi > 0.62) and peripherals (Zi < 2.5 and Pi < 0.62). Besides, the number of module hubs and connectors as well as the OTUs that repeatedly appear in different networks were shown in the corresponding area of the plot.

To ascertain the possible topological role of taxa in the network, the nodes are divided into four categories (i.e., peripherals, connectors, module hubs, and network hubs) according to the within-module connectivity (Zi) and among-module connectivity (Pi) values of taxa (Figure 6). From the Zi-Pi plot, it can be observed that 3.29, 2.39, 1.98, and 2.63% of the nodes in CK, Y10, Y20, and Y40 were classified as module hubs, and 1.14, 0.80, 1.11, and 0.77% of the nodes were classified as connectors, respectively. None of the nodes meet the classification of network hubs. The taxonomy information of keystone taxa (Supplementary Table S5) showed that the members of some phyla such as Proteobacteria, Acidobacteria, Chloroflexi, and Planctomycetes were the highlighted keystone taxa in networks. A total of 8 keystone OTUs appeared in the module hubs and connectors of different networks, namely, OTU102, OTU4, OTU11, OTU2548, OTU358, OTU1329, OTU409, and OTU2943, of which OTU102, OTU4, and OTU11 appeared in the module hubs of CK and Y40 networks, OTU2548 in the module hubs of Y10 networks, OTU409 in the module hubs of Y20 network also appeared in the module hubs of Y40 network, OTU358 in Y10 network connector, and OTU2943 in Y20 network connector appeared in Y40 network connector. According to the taxonomic information of these 8 OTUs (Supplementary Table S6), the members of the Acidobacteria accounted for half, indicating that Acidobacteria played an important role in multiple different networks. While different Acidobacteria members in the connector and module hubs of Y10 network and the module hubs of Y20 network appeared in the module hubs of Y40 network (OTU2548, OTU358, and OTU409), and they did not appear in the module hubs of CK network (Table 6), indicating that tobacco-rice multiple cropping rotation can change the role of Acidobacteria members in the molecular ecological networks.

**Table 6:** Taxonomy information of keystone operational taxonomic units (OTUs) of Acidobacteria members.

	**OTU**	**Domain**	**Phylum**	**Class**	**Order**	**Family**	**Genus**
CK module hubs	OTU81	Bacteria	Acidobacteria	Subgroup11	Unclassified	Unclassified	Unclassified
	OTU20	Bacteria	Acidobacteria	Subgroup4	Pyrinomonadales	Pyrinomonadaceae	RB41
	OTU235	Bacteria	Acidobacteria	Holophagae	Subgroup7	Unclassified	Unclassified
CK Connector	OTU10016	Bacteria	Acidobacteria	Subgroup22	Unclassified	Unclassified	Unclassified
Y10 module hubs	OTU1353	Bacteria	Acidobacteria	Subgroup18	Unclassified	Unclassified	Unclassified
	OTU232	Bacteria	Acidobacteria	Acidobacteriia	Solibacterales	Subgroup3	Bryobacter
	OTU2548	Bacteria	Acidobacteria	Subgroup6	Unclassified	Unclassified	Unclassified
	OTU4772	Bacteria	Acidobacteria	Subgroup6	Unclassified	Unclassified	Unclassified
Y10 Connector	OTU358	Bacteria	Acidobacteria	Subgroup20	Unclassified	Unclassified	Unclassified
	OTU395	Bacteria	Acidobacteria	Subgroup18	Unclassified	Unclassified	Unclassified
	OTU1329	Bacteria	Acidobacteria	Subgroup5	Unclassified	Unclassified	Unclassified
Y20 module hubs	OTU324	Bacteria	Acidobacteria	Acidobacteriia	Solibacterales	Subgroup3	Bryobacter
	OTU409	Bacteria	Acidobacteria	Subgroup22	Unclassified	Unclassified	Unclassified
	OTU1329	Bacteria	Acidobacteria	Subgroup5	Unclassified	Unclassified	Unclassified
Y40 module hubs	OTU409	Bacteria	Acidobacteria	Subgroup22	Unclassified	Unclassified	Unclassified
	OTU358	Bacteria	Acidobacteria	Subgroup20	Unclassified	Unclassified	Unclassified
	OTU8543	Bacteria	Acidobacteria	Subgroup6	Unclassified	Unclassified	Unclassified
	OTU2548	Bacteria	Acidobacteria	Subgroup6	Unclassified	Unclassified	Unclassified
	OTU97	Bacteria	Acidobacteria	Subgroup17	Unclassified	Unclassified	Unclassified

*The OTUs in red indicate that the members play important roles in multiple different networks*.

## Discussion

Paddy-upland multiple cropping rotation can accelerate the soil carbon cycle and the decomposition rate of soil organic matter in the farmland and, therefore, improve soil fertility (Zheng et al., 2016). A previous study has shown that under the condition of planting green manure in the winter fallow period, compared with the double rice cropping system, the paddy-upland multiple cropping rotation significantly increased the soil pH, SOC content, and nitrate nitrogen content and decreased the ammonium nitrogen content (Zhong et al., 2021). Although soil ammonium nitrogen and nitrate nitrogen are the two main forms of nitrogen that can be absorbed by crops, different crops have different preferences for ammonium nitrogen and nitrate nitrogen (Alpha et al., 2009; Wang et al., 2015). In this study, the farmland with the highest soil ammonium nitrogen content (Y20) has a higher grain yield than the farmland with the highest soil nitrate nitrogen content (Y40), suggesting that ammonium nitrogen is more conducive to the increase of grain yield. Understanding the effects of different soil nitrogen nutrient forms on grain yield is crucial for us to develop sustainable agriculture.

The structure and diversity of the soil microbial community are easily affected by factors such as climate change, vegetation types, and soil properties (Liang et al., 2015; Tang et al., 2020; Tan et al., 2021). Previous studies showed that the soil bacterial community structure of paddy fields with different cultivation years of continuous fertilization has differentiated, and the soil bacterial community of paddy fields with 25 years of cultivation is significantly different from that of 9 and 15 years of cultivation (Li et al., 2019a). In this study, we unexpectedly found that there was no significant difference in the diversity and structure of soil bacterial community in tobacco-rice multiple cropping rotation farmlands with different cultivation years. The reason is that tobacco-rice multiple cropping rotation farmlands with different cultivation years has no significant change in soil pH, resulting in no difference in the diversity and structure of bacterial community. Furthermore, since our sampling sites were all in Guiyang County, the homogeneity of geographical location and climatic environment may also cause no difference in the diversity and structure of the bacterial community.

Research has reported that compared with rice monoculture, the composition and diversity of soil bacterial community in paddy-upland multiple cropping rotation have changed (Do Thi et al., 2012), and the taxa of the community, especially the core taxa, play an important role in maintaining the functions of agricultural ecosystems (Shi et al., 2020; Li et al., 2021a). Our results showed that the differences in the nutrient characteristics of farmlands with different cultivation years shaped the differentiation of dominant bacterial genus, and the LEfSe analysis showed that the specific taxa significantly enriched in each treatment were different. Recent studies have shown that Acidobacteria *RB41* plays a key role in control over soil carbon cycle (Stone et al., 2021), and Acidobacteria is involved in the metabolism of inorganic and organic nitrogen sources (Eichorst et al., 2018), and Acidobacteria members can also act as plant growth-promoting rhizobacteria (PGPR) to improve plant growth (Kalam et al., 2020). Our research found that Acidobacteria were highly enriched in farmland with high SOC and ammonium nitrogen content, and the relative abundance of Acidobacteria was significantly positively correlated with pH, SOC, and ammonium nitrogen. Different Acidobacteria subgroups respond differently to soil properties. Some studies have reported that Acidobacteria subgroups 1, 2, and 3 were negatively correlated with soil pH, total C, and N, and Acidobacteria subgroups 4, 6, 7, and 25 were positively correlated with soil pH, total C, and N (Navarrete et al., 2015). Liu et al. (2016) also supported that Acidobacteria subgroups 1, 3, and 13 were negatively correlated with soil pH, while subgroups 4, 6, 7, 11, 17, 18, and 25 were positively correlated with soil pH. In this study, Acidobacteria subgroup 3 was positively correlated with soil pH, and subgroup 17 was negatively correlated with soil pH, which was different from the previous research results. The reason may be the differences in land use and status, covered crops, and geographical regions (Ge et al., 2008; Lu et al., 2019). Our study demonstrated the involvement of Acidobacteria in soil carbon and nitrogen cycle, but we were unable to determine the role of Acidobacteria in soil carbon and nitrogen cycle, and we may need to verify the role of Acidobacteria by isolation and experimental phenotype characterizations in the future.

Studies have proved that the interaction and coexistence patterns between microorganisms have a certain feedback and regulation effect on global climate change, biogeochemical processes, plant growth, and stress resistance (Wang et al., 2019; Mohapatra et al., 2021; Yuan et al., 2021). Our research proves that compared with rice monoculture, tobacco-rice multiple cropping rotation can increase the nodes of the network, and Y40 with the longest cultivation years has the highest network complexity, with most nodes, links, and modules. However, due to the lowest modularity of Y40, its network stability was poor, the interaction between bacteria was more vulnerable to the interference of external environmental stress (Hernandez et al., 2021), and the weaker bacterial community stability and resistance may be the reason for the decline of grain yield in Y40 farmland (Horner et al., 2019). Moreover, we found that the increase of soil ammonium nitrogen content strengthened the competitive relationship between bacterial interactions, and more competitive relationships between bacterial interactions will enhance the stability of the community (Coyte et al., 2015; Ghoul and Mitri, 2016). The better the soil nutrient conditions, the stronger the competitive relationship between bacterial interactions has also been confirmed (Wang et al., 2018). By classifying the keystone taxa in the molecular ecological network, we found that there were 8 repeated OTUs in different networks, of which 4 OTUs were Acidobacteria. We hold the opinion that Acidobacteria plays an important role in all networks. In addition, tobacco-rice multiple cropping rotation farmlands with different cultivation years have the same keystone OTUs of Acidobacteria members, and these OTUs are inconsistent with rice monoculture farmland, which indicates that tobacco-rice multiple cropping rotation has changed the Acidobacteria members with important functions in the network.

The cultivation years of the farmland affected the richness of the bacterial community, and the increase of cultivation age reduced the diversity of the bacterial community and changed the community structure and composition (Chang et al., 2021; Tong et al., 2021). Continuous cropping has caused the aggravation of soil-borne diseases in farmland, resulting in a decline in crop quality and yield, and severely restricted the productivity and sustainable development of farmland ecosystems (Arafat et al., 2019; Gao et al., 2020; Tian et al., 2020). However, recent studies have shown that continuous cultivation of farmland has no significant negative impact on crop yield, which is attributed to the use of organic fertilizers and crop straw mulching return (Rannestad and Gessesse, 2020). With the increase of cultivation years, the diversity and structure of bacterial communities did not change significantly in tobacco-rice multiple cropping rotation farmlands in this study. Compared with the farmland with 10–20 years of cultivation, the grain yield of farmland with 20–40 years of cultivation has increased significantly. These studies prove that under the tobacco-rice multiple cropping rotation system in this study, there are no continuous cropping obstacles due to reasonable nutrient planning, agronomic management, and crop straw returning (Chen et al., 2018). However, the decline of soil bacterial community stability and grain yield in farmland with cultivation years greater than 40 years indicates that excessive cultivation years are not conducive to the sustainable use of soil and the stable production of crops. Our research provides a theoretical basis for rationally developing crop planting patterns, resisting continuous cropping obstacles, and stabilizing crop production and has important practical significance for guiding sustainable agricultural production.

## Conclusion

Tobacco-rice multiple cropping rotation improved soil fertility and the diversity of the bacterial community and finally increased crop yields. The farmland with the highest SOC and ammonium nitrogen content enriched Acidobacteria members. Different Acidobacteria subgroups respond differently to changes in soil properties, and Acidobacteria members play a key role in maintaining biodiversity and the local ecosystem. The decrease in the stability of bacterial communities in farmland where the cultivation period is too long may be the reason for the decrease in crop yield. Thus, choosing reasonable crop planting patterns according to local conditions and reducing the years of planting crops in the same farmland are more conducive to the sustainable development and production of farmland and crops.

## Data Availability Statement

The datasets presented in this study can be found in online repositories. The names of the repository/repositories and accession number(s) can be found below: the raw sequencing data can be found at the National Centre for Biotechnology Information (NCBI) Sequence Read Archive (SRA) (Accession Number: PRJNA775899).

## Author Contributions

TC: carried out the experiments and wrote the article. RH: formal analysis and wrote the article. ZZ, JY, HF, XD, and WY: carried out the experiments. QW: methodology. SP: methodology and formal analysis. JL: methodology, formal analysis, and wrote the article. All authors contributed to the article and approved the submitted version.

## Funding

This study was supported by the Natural Science Foundation of Hunan Province (2020JJ4038 and 2020JJ4374) and the Key Project of Science and Technology of Hunan Tobacco Company Chenzhou Branch (CYKJ2018-05 and CZYC2021JS10).

## Conflict of Interest

TC, XD and WY were employed by the Hunan Tobacco Company. This study received funding from Key Project of Science and Technology of Hunan Tobacco Company Chenzhou Branch and Natural Science Foundation of Hunan Province. The funder had involvement with the study design and data collection. All authors declare no other competing interests.

## Publisher's Note

All claims expressed in this article are solely those of the authors and do not necessarily represent those of their affiliated organizations, or those of the publisher, the editors and the reviewers. Any product that may be evaluated in this article, or claim that may be made by its manufacturer, is not guaranteed or endorsed by the publisher.
