# Supplementary material for: Soil Bacterial Community in the Multiple Cropping System Increased Grain Yield Within 40 Cultivation Years
Source: Front Plant Sci. 2021 Dec 20;12:804527. doi: 10.3389/fpls.2021.804527 (PMC8721226; doi:10.3389/fpls.2021.804527)
Supplement: Supplementary file 1 [file Data_Sheet_1.docx]

**Soil bacterial community in multiple cropping system increased grain yield within 40 cultivation years**

Tao Chen ^a,b,^^[[1]](#footnote-1)^, Ruiwen Hu ^a,1^, Zhongyi Zheng ^a^, Jiayi Yang ^a^, Huan Fan ^a^, Xiaoqiang Deng ^b^, Wang Yao ^c^, Qiming Wang ^d^, Shuguang Peng ^e,*^, Juan Li ^a,*^

^a^ *College of Agronomy, Hunan Agricultural University, Changsha, China*

^b^ *Hunan Tobacco Company Chenzhou Branch, Chenzhou, China*

^c^ *Hunan Tobacco Company Zhangjiajie Branch, Zhangjiajie, China*

^d^ *College of Bioscience and Biotechnology, Hunan Agricultural University, Changsha, China*

^e^ *China National Tobacco Corporation Hunan Branch, Changsha, China*


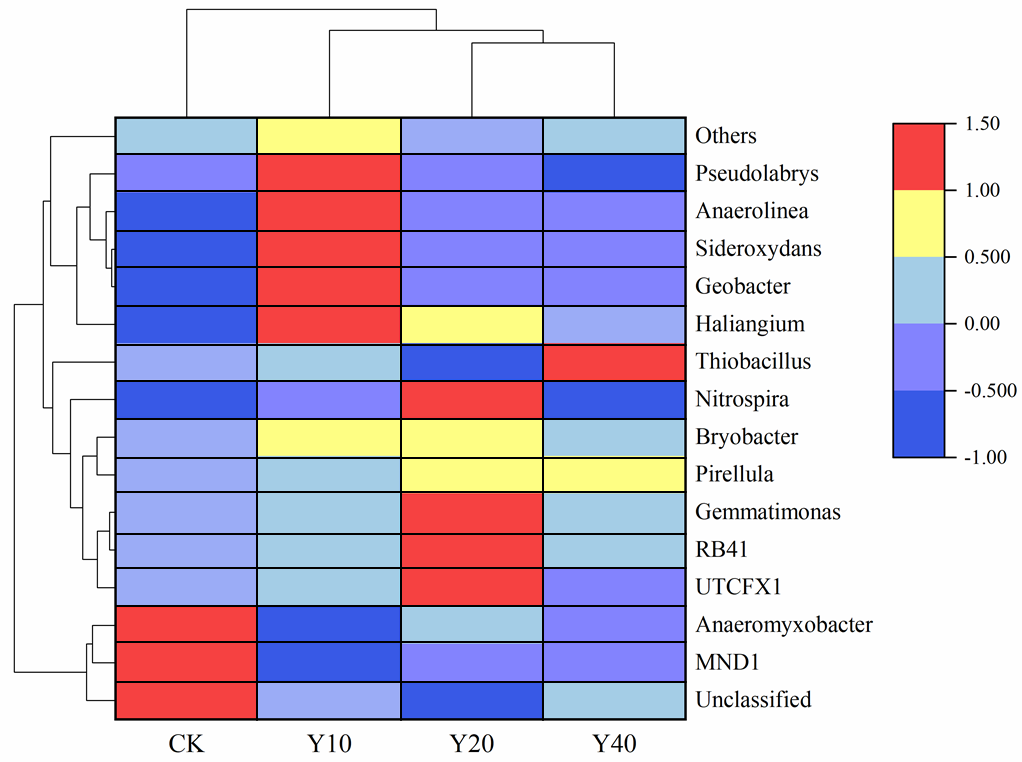


**Fig.S1.** Cluster heat map of 15 most abundant bacterial genera in the soil bacterial community. Results are means of eight replicates.


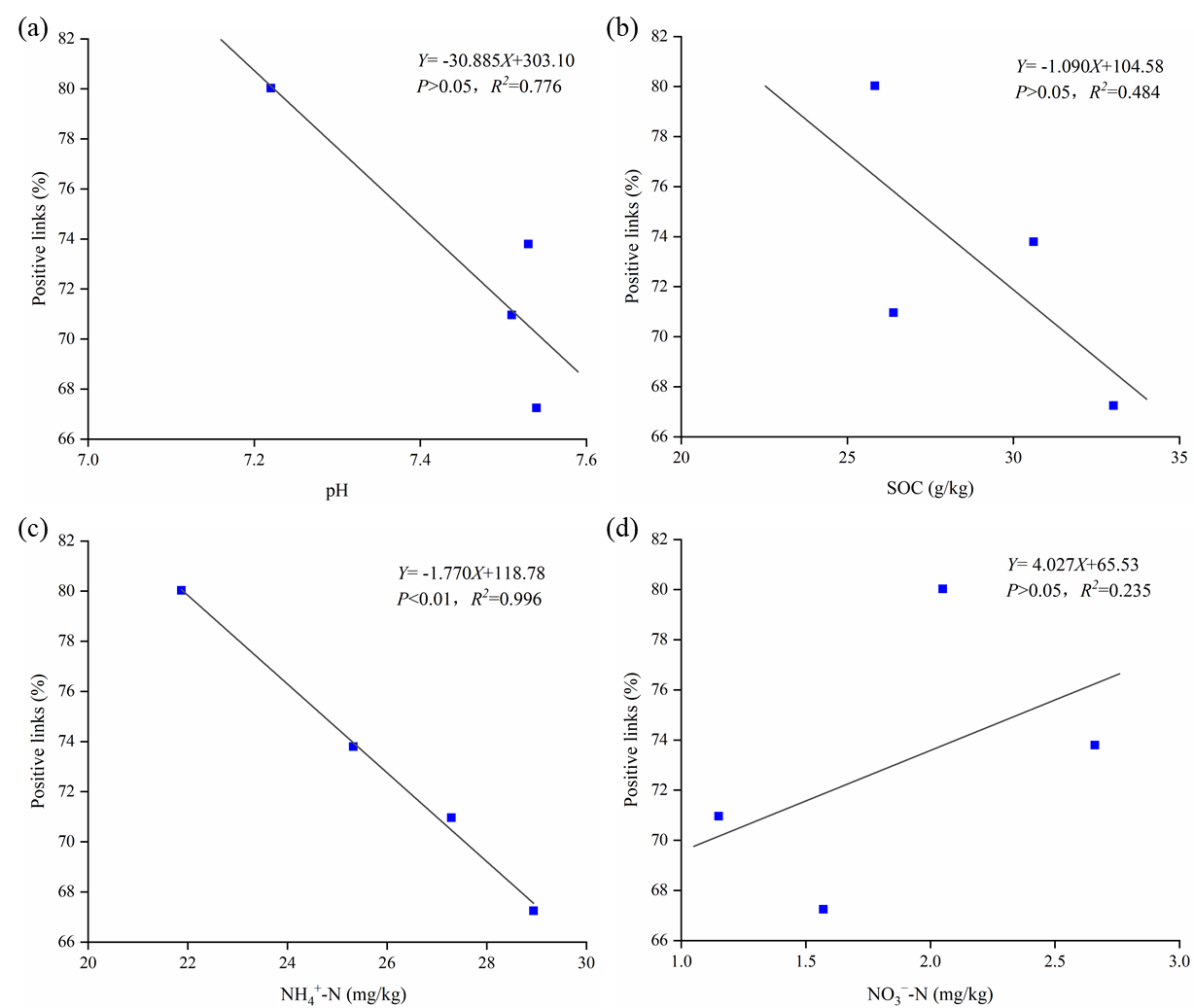


**Fig.S2.** Linear regression between percentage of positive links of molecular ecological network and edaphic factors. (a) positive links and pH. (b) positive links and SOC. (c) positive links and NH_4_^+^-N. (d) positive links and NO_3_^−^-N.

**Table.S1.** Dissimilarity analysis of soil bacterial community structure.

| Treatment | ADONIS | | ANOSIM | | MRPP | |
| --- | --- | --- | --- | --- | --- | --- |
|  | R^2^ | *P* | R | *P* | δ | *P* |
| CK vs Y10 | 0.133 | **0.034** | 0.087 | 0.162 | 0.526 | **0.01** |
| CK vs Y20 | 0.156 | **0.014** | 0.272 | **0.008** | 0.473 | **0.008** |
| CK vs Y40 | 0.096 | 0.138 | 0.086 | 0.118 | 0.525 | 0.093 |
| Y10 vs Y20 | 0.110 | 0.109 | 0.121 | 0.099 | 0.427 | 0.079 |
| Y10 vs Y40 | 0.081 | 0.258 | 0.045 | 0.277 | 0.479 | 0.188 |
| Y20 vs Y40 | 0.092 | 0.165 | 0.065 | 0.179 | 0.425 | 0.117 |

Note: The bold numbers indicate significant correlation at the level of *p* < 0.05.

**Table.S2.** An envfit analysis (envfit function used with 999 permutations) shows soil properties variables that correlate to bacterial community.

| Variables | RDA1 | RDA2 | R^2^ | *p* |
| --- | --- | --- | --- | --- |
| pH | 0.87754 | 0.47950 | 0.2873 | **0.004** |
| SOC | 0.79541 | -0.60608 | 0.1302 | 0.144 |
| NH_4_^+^-N | 0.86551 | 0.50089 | 0.3062 | **0.005** |
| NO_3_^−^-N | -0.73268 | -0.68058 | 0.1892 | **0.048** |

Note: The bold numbers indicate significant correlation at the level of *p* < 0.05. SOC, Soil organic carbon; NH_4_^+^-N, Ammonium-N; NO_3_^−^-N, Nitrate-N.

**Table.S3.** The proportion of all variables explained the bacterial community variation.

|  | Inertia | Proportion |
| --- | --- | --- |
| Total | 6.444 | 100% |
| Constrained | 0.965 | 14.98% |
| Unconstrained | 5.479 | 85.02% |

**Table.S4.** LDA Effect Size analysis results (only with significant differences).

| Treatment | Domain | Phylum | Class | Order | Family | Genus | LDA score | *P* value |
| --- | --- | --- | --- | --- | --- | --- | --- | --- |
| Y40 | Bacteria | Acidobacteria | Subgroup_5 | - | - | - | 3.132 | 0.002 |
| Y40 | Bacteria | Acidobacteria | Subgroup_5 | Unclassified | - | - | 3.132 | 0.002 |
| Y40 | Bacteria | Acidobacteria | Subgroup_5 | Unclassified | Unclassified | - | 3.132 | 0.002 |
| Y40 | Bacteria | Acidobacteria | Subgroup_5 | Unclassified | Unclassified | Unclassified | 3.132 | 0.002 |
| Y40 | Bacteria | Armatimonadetes | - | - | - | - | 3.141 | 0.004 |
| Y40 | Bacteria | Proteobacteria | Deltaproteobacteria | Myxococcales | Polyangiaceae | - | 3.144 | 0.035 |
| Y40 | Bacteria | Proteobacteria | Gammaproteobacteria | Betaproteobacteriales | Hydrogenophilaceae | - | 3.396 | 0.003 |
| Y40 | Bacteria | Proteobacteria | Gammaproteobacteria | Betaproteobacteriales | Hydrogenophilaceae | Thiobacillus | 3.370 | 0.002 |
| Y20 | Bacteria | Acidobacteria | - | - | - | - | 4.258 | 0.039 |
| Y20 | Bacteria | Acidobacteria | Blastocatellia__Subgroup_4_ | - | - | - | 4.047 | 0.002 |
| Y20 | Bacteria | Acidobacteria | Blastocatellia__Subgroup_4_ | 11_24 | - | - | 3.546 | 0.001 |
| Y20 | Bacteria | Acidobacteria | Blastocatellia__Subgroup_4_ | 11_24 | Unclassified | - | 3.546 | 0.001 |
| Y20 | Bacteria | Acidobacteria | Blastocatellia__Subgroup_4_ | 11_24 | Unclassified | Unclassified | 3.546 | 0.001 |
| Y20 | Bacteria | Acidobacteria | Blastocatellia__Subgroup_4_ | Pyrinomonadales | - | - | 3.829 | 0.008 |
| Y20 | Bacteria | Acidobacteria | Blastocatellia__Subgroup_4_ | Pyrinomonadales | Pyrinomonadaceae | - | 3.829 | 0.008 |
| Y20 | Bacteria | Acidobacteria | Blastocatellia__Subgroup_4_ | Pyrinomonadales | Pyrinomonadaceae | RB41 | 3.829 | 0.008 |
| Y20 | Bacteria | Acidobacteria | Holophagae | - | - | - | 3.556 | 0.031 |
| Y20 | Bacteria | Acidobacteria | Holophagae | Subgroup_7 | - | - | 3.552 | 0.033 |
| Y20 | Bacteria | Acidobacteria | Holophagae | Subgroup_7 | Unclassified |  | 3.544 | 0.033 |
| Y20 | Bacteria | Acidobacteria | Holophagae | Subgroup_7 | Unclassified | Unclassified | 3.544 | 0.033 |
| Y20 | Bacteria | Chloroflexi | Anaerolineae | SBR1031 | Unclassified |  | 3.412 | 0.024 |
| Y20 | Bacteria | Chloroflexi | Anaerolineae | SBR1031 | Unclassified | Unclassified | 3.412 | 0.024 |
| Y20 | Bacteria | Gemmatimonadetes | S0134_terrestrial_group | - | - | - | 3.227 | 0.000 |
| Y20 | Bacteria | Gemmatimonadetes | S0134_terrestrial_group | Unclassified | - | - | 3.227 | 0.000 |
| Y20 | Bacteria | Gemmatimonadetes | S0134_terrestrial_group | Unclassified | Unclassified | - | 3.227 | 0.000 |
| Y20 | Bacteria | Gemmatimonadetes | S0134_terrestrial_group | Unclassified | Unclassified | Unclassified | 3.227 | 0.000 |
| Y10 | Bacteria | Acidobacteria | Acidobacteriia | - | - | - | 3.603 | 0.031 |
| Y10 | Bacteria | Acidobacteria | Acidobacteriia | Acidobacteriales | - | - | 3.152 | 0.040 |
| Y10 | Bacteria | Acidobacteria | Blastocatellia__Subgroup_4_ | Blastocatellales | - | - | 3.170 | 0.047 |
| Y10 | Bacteria | Acidobacteria | Blastocatellia__Subgroup_4_ | Blastocatellales | Blastocatellaceae | - | 3.170 | 0.047 |
| Y10 | Bacteria | Chloroflexi | Anaerolineae | Anaerolineales | Anaerolineaceae | RBG_16_58_14 | 3.231 | 0.013 |
| Y10 | Bacteria | Patescibacteria | Microgenomatia | Candidatus_Woesebacteria | - | - | 3.137 | 0.037 |
| Y10 | Bacteria | Patescibacteria | Microgenomatia | Candidatus_Woesebacteria | Unclassified | - | 3.089 | 0.015 |
| Y10 | Bacteria | Patescibacteria | Microgenomatia | Candidatus_Woesebacteria | Unclassified | Unclassified | 3.089 | 0.015 |
| Y10 | Bacteria | Proteobacteria | Gammaproteobacteria | Betaproteobacteriales | Gallionellaceae | Sideroxydans | 3.488 | 0.033 |
| Y10 | Bacteria | Proteobacteria | Gammaproteobacteria | Ectothiorhodospirales | - | - | 3.169 | 0.001 |
| Y10 | Bacteria | Proteobacteria | Gammaproteobacteria | Ectothiorhodospirales | Thioalkalispiraceae | - | 3.168 | 0.001 |
| Y10 | Bacteria | Proteobacteria | Gammaproteobacteria | Ectothiorhodospirales | Thioalkalispiraceae | Thioalkalispira | 3.168 | 0.001 |
| Y10 | Bacteria | Verrucomicrobia | - | - | - | - | 3.420 | 0.012 |
| Y10 | Bacteria | Verrucomicrobia | Verrucomicrobiae | - | - | - | 3.420 | 0.012 |
| Y10 | Bacteria | Verrucomicrobia | Verrucomicrobiae | Pedosphaerales | - | - | 3.276 | 0.018 |
| Y10 | Bacteria | Verrucomicrobia | Verrucomicrobiae | Pedosphaerales | Pedosphaeraceae | - | 3.276 | 0.018 |
| CK | Bacteria | Acidobacteria | Subgroup_17 | - | - | - | 3.275 | 0.024 |
| CK | Bacteria | Acidobacteria | Subgroup_17 | Unclassified | - | - | 3.275 | 0.024 |
| CK | Bacteria | Acidobacteria | Subgroup_17 | Unclassified | Unclassified | - | 3.275 | 0.024 |
| CK | Bacteria | Acidobacteria | Subgroup_17 | Unclassified | Unclassified | Unclassified | 3.275 | 0.024 |
| CK | Bacteria | Proteobacteria | Deltaproteobacteria | Myxococcales | Archangiaceae | - | 3.396 | 0.019 |
| CK | Bacteria | Proteobacteria | Deltaproteobacteria | Myxococcales | Archangiaceae | Anaeromyxobacter | 3.400 | 0.023 |
| CK | Bacteria | Rokubacteria | - | - | - | - | 3.473 | 0.036 |
| CK | Bacteria | Rokubacteria | NC10 | - | - | - | 3.473 | 0.036 |
| CK | Bacteria | Rokubacteria | NC10 | Rokubacteriales | - | - | 3.431 | 0.026 |
| CK | Bacteria | Rokubacteria | NC10 | Rokubacteriales | Unclassified | - | 3.431 | 0.026 |
| CK | Bacteria | Rokubacteria | NC10 | Rokubacteriales | Unclassified | Unclassified | 3.431 | 0.026 |

**Table.S5.** Taxonomy of keystone OTUs in *Zi*-*Pi* plot. Module hubs: *Z_i_* > 2.5, connectors: *P_i_* > 0.62. P: Phylum. The OTUs in red indicate that the species play important roles in multiple different networks.

|  | Nodes | Taxonomy |
| --- | --- | --- |
| CK Module Hubs | OTU302, OTU2560, OTU264, OTU601, OTU18, OTU102, OTU8, OTU166, OTU4, OTU120 | P: Proteobacteria |
|  | OTU134 | P: Planctomycetes |
|  | OTU81, OTU20, OTU235 | P: Acidobacteria |
|  | OTU52, OTU675, OTU412, OTU396 | P: Chloroflexi |
|  | OTU87, OTU11 | P: Nitrospirae |
|  | OTU197 | P: Latescibacteria |
|  | OTU428 | P: Gemmatimonadetes |
|  | OTU391 | P: Dadabacteria |
| CK Connector | OTU10016 | P: Acidobacteria |
|  | OTU7790, OTU226, OTU1312 | P: Proteobacteria |
|  | OTU853, OTU2630, OTU6873 | P: Chloroflexi |
|  | OTU453 | P: Actinobacteria |
| Y10 Module Hubs | OTU891, OTU1106, OTU482, OTU1830, OTU696 | P: Proteobacteria |
|  | OTU312, OTU961 | P: Bacteroidetes |
|  | OTU683, OTU1060 | P: Planctomycetes |
|  | OTU1353, OTU232, OTU2548, OTU4772 | P: Acidobacteria |
|  | OTU284, OTU16921, OTU279 | P: Chloroflexi |
|  | OTU1682 | P: Latescibacteria |
|  | OTU185 | P: Rokubacteria |
|  | OTU1187 | P: Gemmatimonadetes |
|  | OTU393 | P: Zixibacteria |
|  | OTU2250 | P: Nitrospirae |
| Y10 Connector | OTU100, OTU313 | P: Proteobacteria |
|  | OTU358, OTU395, OTU1329 | P: Acidobacteria |
|  | OTU3 | P: Thaumarchaeota |
|  | OTU48 | P: Chloroflexi |
| Y20 Module Hubs | OTU648, OTU1315, OTU4 | P: Proteobacteria |
|  | OTU16993, OTU288, OTU1607, OTU172 | P: Chloroflexi |
|  | OTU371 | P: Bacteroidetes |
|  | OTU1653, OTU1888 | P: Verrucomicrobia |
|  | OTU324, OTU409, OTU1329 | P: Acidobacteria |
|  | OTU238 | P: Armatimonadetes |
|  | OTU241, OTU3399 | P: Planctomycetes |
| Y20 Connector | OTU162 | P: Proteobacteria |
|  | OTU32 | P: Nitrospirae |
|  | OTU2943, OTU8886, OTU8335, OTU216, OTU1248 | P: Chloroflexi |
|  | OTU10 | P: Crenarchaeota |
|  | OTU310 | P: Cyanobacteria |
| Y40 Module Hubs | OTU21, OTU102, OTU65, OTU22, OTU1335, OTU909, OTU4 | P: Proteobacteria |
|  | OTU170, OTU321 | P: Gemmatimonadetes |
|  | OTU512 | P: Latescibacteria |
|  | OTU26, OTU2943, OTU627, OTU430, OTU666, OTU340 | P: Chloroflexi |
|  | OTU11 | P: Nitrospirae |
|  | OTU409, OTU358, OTU8543, OTU2548, OTU97 | P: Acidobacteria |
|  | OTU227, OTU4874 | P: Planctomycetes |
| Y40 Connector | OTU323, OTU188, OTU291 | P: Proteobacteria |
|  | OTU569 | P: Spirochaetes |
|  | OTU156 | P: Planctomycetes |
|  | OTU1637 | P: Patescibacteria |
|  | OTU686 | P: Zixibacteria |

**Table.S6.** Taxonomy information of OTUs marked red.

| OTU | Domain | Phylum | Class | Order | Family | Genus |
| --- | --- | --- | --- | --- | --- | --- |
| OTU102 | Bacteria | Proteobacteria | Deltaproteobacteria | NB1-j | Unclassified | Unclassified |
| OTU4 | Bacteria | Proteobacteria | Alphaproteobacteria | Rhizobiales | Xanthobacteraceae | Pseudolabrys |
| OTU11 | Bacteria | Nitrospirae | Thermodesulfovibrionia | Unclassified | Unclassified | Unclassified |
| OTU2548 | Bacteria | Acidobacteria | Subgroup6 | Unclassified | Unclassified | Unclassified |
| OTU358 | Bacteria | Acidobacteria | Subgroup20 | Unclassified | Unclassified | Unclassified |
| OTU1329 | Bacteria | Acidobacteria | Subgroup5 | Unclassified | Unclassified | Unclassified |
| OTU409 | Bacteria | Acidobacteria | Subgroup22 | Unclassified | Unclassified | Unclassified |
| OTU2943 | Bacteria | Chloroflexi | Anaerolineae | Anaerolineales | Anaerolineaceae | Unclassified |

1. ^*^ Corresponding author.

   E-mail address: [kyspsg@sina.com](mailto:kyspsg@sina.com) (SG. Peng), adalee619@163.com (J. Li).

   These authors contributed equally to this work. [↑](#footnote-ref-1)
